# Synergistic Interaction between 5-FU and an Analog of Sulforaphane—2-Oxohexyl Isothiocyanate—In an In Vitro Colon Cancer Model

**DOI:** 10.3390/molecules26103019

**Published:** 2021-05-19

**Authors:** Małgorzata Milczarek, Anna Pogorzelska, Katarzyna Wiktorska

**Affiliations:** Department of Drug Biotechnology and Bioinformatics, National Medicines Institute, 30/34 Chełmska St., 00-725 Warszawa, Poland; a.pogorzelska@nil.gov.pl

**Keywords:** 5-fluorouracil, isothiocyanates, sulforaphane, synergistic effect

## Abstract

Combination therapy is based on the beneficial effects of pharmacodynamic interaction (synergistic or additive) between combined drugs or substances. A considerable group of candidates for combined treatments are natural compounds (e.g., isothiocyanates) and their analogs, which are tested in combination with anticancer drugs. We tested the anticancer effect of the combined treatment of isothiocyanate 2-oxohexyl isothiocyanate and 5-fluorouracil in colon and prostate cancer cell lines. The type of interaction was described using the Chou-Talalay method. The cytostatic and cytotoxic activities of the most promising combined treatments were investigated. In conclusion, we showed that combined treatment with 5-fluorouracil and 2-oxohexyl isothiocyanate acted synergistically in colon cancer. This activity is dependent on the cytostatic properties of the tested compounds and leads to the intensification of their individual cytotoxic activity. The apoptotic process is considered to be the main mechanism of cytotoxicity in this combined treatment.

## 1. Introduction

A cycle-specific antimetabolite, 5-fluorouracil (5-FU) is an antineoplastic chemotherapeutic agent that is widely used in the treatment of colorectal cancer (CRC). It was introduced into clinical use over 50 years ago; however, despite progress in novel cancer therapies, it remains the mainstay of systemic colorectal cancer treatment and the main constituent of chemotherapy combination regimens [1]. Despite the encouraging progress in CRC therapy, the efficacy and safety of 5-FU are still inadequate. Drug resistance as well as toxic side effects (e.g., increased risks of infection, anemia, and diarrhea) are the main obstacles to the successful treatment of colon cancer [1,2]. For this reason, several 5-FU-based combination regimens have been proposed to increase antitumor activity, reduce the side effects, and overcome clinical resistance. 

One example is leucovorin (LV; folinic acid), which inhibits the side effects of 5-FU and potentiates its anticancer efficacy. LV administered together with 5-FU has been shown to improve patient survival and tumor response rate; such administration has become a standard 5-FU-based regimen [3]. For increased efficacy, 5-FU is frequently combined with oxaliplatin and irinotecan, and such combined therapies (with LV) have become established as regimens for the treatment of CRC, resulting in an improved survival rate of approximately two years [4,5]. 

Combination therapy is a cornerstone of cancer chemotherapy and is based on the beneficial effects of pharmacodynamic interaction (synergistic or additive) between combined drugs or substances [6]. Synergistic interactions lead to more effective therapies than in the case of monotherapies. Combined treatments based on compounds acting in a synergistic or an additive manner exhibit less toxicity than monotherapies since they are applied in lower doses and interact with different molecules or pathways [7]. 

A considerable group of candidates for combined treatments are natural compounds, e.g., resveratrol, genistein, and sulforaphane. As a result of their anticancer activity, they are intensively tested to potentiate the effectiveness of conventional chemotherapy drugs. Moreover, these compounds are considered to be modifiers of chemotherapy burden, e.g., curcumin counteracts the ototoxicity of cisplatin and sulforaphane attenuates the cardiotoxicity of doxorubicin [8,9].

In combination with phytochemicals, 5-FU is one of the most widely investigated chemotherapy drugs. It has been tested in combination with curcumin, resveratrol, *Ganoderma lucidum* polysaccharides, genistein, geraniol, betulinic acid, gossypol, and protopanaxadiol. In addition, a combination of geraniol, protopanaxadiol, and 5-FU has been shown to reduce tumor volume in in vivo models [10,11,12,13,14,15,16,17]. In all cases, combination treatment synergistically induced apoptosis in colon cancer cells. However, the observed synergistic effect could be the result of the combined impact on autophagy. Autophagy is a catabolic process exerted in cells in response to stressful conditions, e.g., to nutrient deprivation or damage to proteins or DNA. If the process is too intense, it can trigger cell death. It was shown that 5-FU combined with *Phellinus linteus* or sulforaphane elicited autophagic cell death in triple-negative breast cancer cells [18,19]. In the case of colon cancer, simultaneous apoptosis and autophagic cell death were observed, which could be regarded as an antagonistic interaction caused by the cytoprotective capability of autophagy [20]. On the other hand, inhibition of autophagy and induction of apoptosis were proposed to be the basis of synergy in the case of the combined 5-FU and tangeretin treatment in colon cells [21].

**Figure 1:**
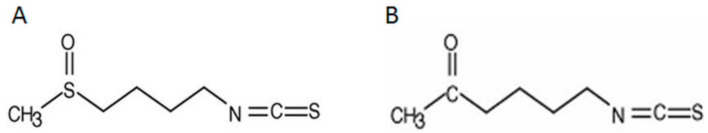
Structural formulae of isothiocyanates: (**A**) sulforaphane; (**B**) 2-oxohexyl isothiocyanate.

Sulforaphane is one of the most commonly studied phytocompounds. Its anticancer and protective properties have been proven in both in vitro and in vivo models. Currently, it is most frequently assessed in clinical studies, especially for treating prostate cancer patients and chemoprevention in this disease. Our previous research showed that sulforaphane attenuates anticancer activity in breast and colon cancer cells and exhibits better activity than its analog, alyssin, which has a prolonged carbon chain [19,22]. In this paper, we describe 5-FU in combination with another sulforaphane analog, 2-oxohexyl isothiocyanate (2-oxohexyl ITC), which has a different structural modification (see Figure 1) and demonstrated relevant anticancer properties in previous studies. 

The anticancer activity of 2-oxohexyl ITC was demonstrated in L-120 leukemia cancer cells, ME-18 melanoma cells, and in BRCA1 mutated cell lines. It induces apoptosis through the mitochondrial pathway and cell cycle block [23,24]. Additionally, the studies conducted on L-120 leukemia cancer cells and ME-18 melanoma cells showed that 2-oxohexyl ITC exhibited more potent anticancer capabilities than sulforaphane [24]. However, the combined treatment of 2-oxohexyl ITC and anticancer drugs has not been investigated in cancer cell lines. Our previous study on the effect of 5-FU and 2-oxohexyl ITC on normal cells provided promising results since the cytotoxicity of 5-FU was not elevated [25]. 

Given the anticancer activity of 5-FU and 2-oxohexyl ITC, we examined the effect and mechanism of action of their combination in colon and prostate cancer cells, which are the third and fourth most commonly occurring malignancies. The results of this study on the anticancer properties of 5-FU in combination with 2-oxohexyl ITC show the synergistic anticancer activity of this combined treatment, particularly in colon cancer cell lines. Apoptosis induction rather than autophagy was shown to be the major mechanism of cell death.

## 2. Results and Discussion 

### 2.1. Cell Growth Inhibition and Interaction Type 

**Figure 2:**
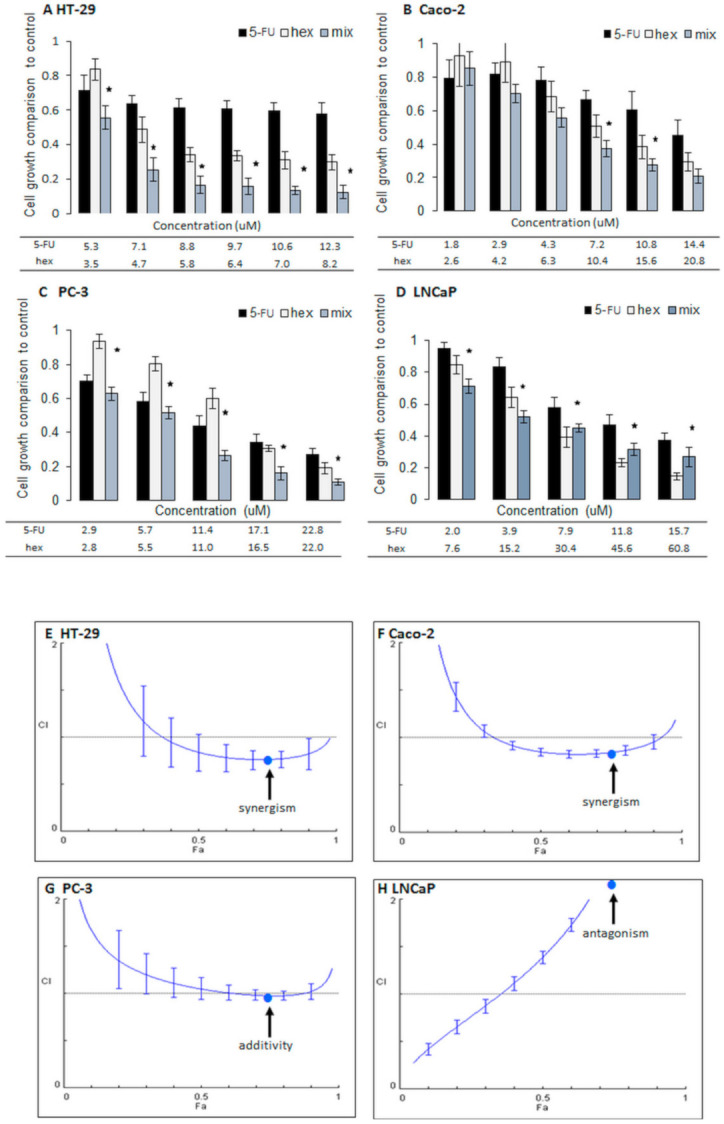
Cell growth inhibition and interaction types. The changes in cell growth of cancer cell lines after the administration of compounds alone and in combination: 5-fluorouracil (5-FU) with 2-oxohexyl ITC (hex) in HT-29 (**A**), Caco-2 (**B**), PC-3 (**C**), and LNCaP (**D**) cell lines. * The cell growth values after the administration of a combination treatment were statistically significantly different from the cell growth values after administrations of 2-oxohexyl ITC and 5-FU alone; *p* < 0.05. Cell growth was determined using the MTT method. Combination treatment: the cells were incubated with 2-oxohexyl ITC for 24 h and then with 5-FU for 72 h. In individual administrations, one component of the combination was used. Combination index values (CI) depending on the fraction affected (fa) in HT-29 (**E**) and Caco-2 (**F**) colon cancer cell lines and in PC-3 (**G**) and LNCaP (**H**) prostate cancer cell lines for 5-FU administration with 2-oxohexyl ITC. CI > 1 = antagonism; CI < 1 = synergism; CI ± 1.0 = an additive effect. The Chou-Talalay method was used to determine the CI. The arrows indicate the type of interaction for fa = 0.75.

**Figure 3:**
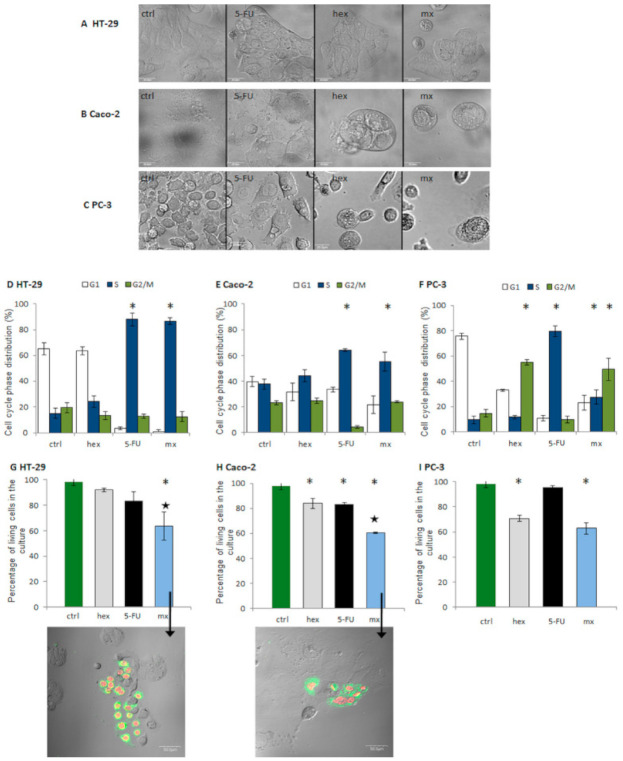
Morphological changes in the HT-29 (**A**), Caco-2 (**B**), and PC-3 (**C**) cells treated with 5-FU, hex, and the combined treatment (mx). The changes in distribution of the cell cycle phases in colon and prostate cancer cell lines when compared to the control after the administration of the combined treatment and the individual compounds in the HT-29 (**D**), Caco-2 (**E**), and PC-3 (**F**) cell lines. Cell cycle distribution was determined by flow cytometry. Combination treatment: the cells were incubated with 2-oxohexyl ITC for 24 h and then with 5-FU for 72 h. In individual administrations, one component of the combination was used. The changes of the living cells percentage in the cell culture after the administration of individual compounds and the combined treatment in the Caco-2 (**G**), HT-29 (**H**), and PC-3 (**I**) cell lines. Large black star—statistically significant difference between the percentage of living cells after the administration of the combined treatment and after administrations of 2-oxohexyl ITC and 5-FU alone. * Statistically significant difference from the control; *p* < 0.05. Cell viability was determined using the FDA/PI method using flow cytometry. Apoptosis detection in HT-29 colon cancer and Caco-2 cancer cell lines is indicated by arrows in graphs (**G**,**H**). Green—cell membrane stained with Annexin V-FITC. Red—nuclei stained with PI.

Cell growth was determined after combination treatment and individual treatment using an MTT assay in prostate cancer cell lines LNCaP and PC-3 and in colon cancer cell lines HT-29 and Caco-2 (Figure 2). On the basis of these results, the type of interaction was defined using the Chou-Talalay method (Figure 3) [26]. The most prominent effect was observed after combination treatment in HT-29 cells, which represent aggressive colon cancer cells [27]. At most combined concentrations, cell growth was reduced to a value below 0.5 (fa > 0.5; fa—fraction of the cells affected) in comparison to the control; untreated cells and the combined treatment were significantly more potent than individual treatments. In those cases, the combination of 5-FU and 2-oxohexyl ITC reduced cell growth by half in comparison to the individual treatments (Figure 2B). This effect was the synergistic interaction between 5-FU and 2-oxohexyl ITC according to the Chou-Talalay analysis of drug–drug interaction. Except the lowest concentration, the synergistic interaction occurred between 5-FU and 2-oxohexyl ITC. The calculated CI approximated 0.8 for fa above 0.5, which generated a more prominent effect than in the individual treatments.

In the Caco-2 colon cancer cells, the effect of the combined treatment was weaker (Figure 2A). It was only at fa = 0.6 or 0.7 that the combined treatments had a significantly stronger impact on cell growth than the individual treatments. The calculated CIs were lower than 1 for most of the studied combinations and, although slightly weaker, the effects were similar to those observed in the HT-29 cells. An additive effect was observed with fa = 0.9 and the CI close to 1, but this was an exception.

In PC-3 cells, which represent an aggressive and hormone-resistant prostate cancer cell type, the administration of 2-oxohexyl ITC with 5-FU led to an approximate 10% decrease in cell growth in comparison to the individual treatments. Combined treatments at the highest concentrations resulted in an 80% drop in cell number. In these cases, the combined effect was about 50% stronger than the effect of the individual treatments. In these cells, an additive effect was observed when fa was higher than 0.4, with the lowest value being 1 at fa = 0.8, producing an 80% drop in cell number.

In hormone-sensitive LNCaP cells, for low concentrations when cell growth was reduced to less than 50%, the results of combined treatments were insignificantly (approximately 10%) stronger than after 2-oxohexyl ITC treatment alone. On the contrary, when cell growth was reduced by more than 50% in comparison to the control, the effects of combined 5-FU and 2-oxohexyl ITC treatments were weaker than the effects of 2-oxohexyl ITC treatment alone. As expected, these opposing effects were the results of the antagonistic interaction between 5-FU and 2-oxohexyl ITC calculated for a fa higher than 0.4. For these concentrations of components, a CI higher than 1.1 (e.g., at fa = 0.75 and a CI value of around 2.8) was observed. As demonstrated, antagonism indicates weaker anticancer activity for the combined treatment than the individual treatment; hence, such phenomena exclude the drug candidate from further study.

The results described above are consistent with our earlier findings. We proved that sulforaphane and its analog (alyssin) acted more efficiently in combination with 5-FU in colon cancer cells than in prostate cancer cells [22]. Moreover, the results indicate that the combination of 5-FU and the sulforaphane analog is mostly effective in hormone-independent (PC-3) and aggressive types of cancer cells (PC-3 and HT-29), which is also in agreement with our previous results concerning the interaction between doxorubicin and sulforaphane in breast cancer cells. Other authors also demonstrated that isothiocyanates can interact synergistically with anticancer drugs in androgen-independent prostate cancer cell lines, e.g., 2-phenylethyl isothiocyanate combined with doxorubicin in PC-3 cells and sulforaphane with Taxol and cisplatin in PC-3 and DU145 [28,29]. In LNCaP androgen-dependent cells, isothiocyanates applied in combination with anticancer drugs did not act synergistically. Interestingly, other phytochemicals, e.g., fisetin, or small-molecule inhibitors, e.g., YK-4-279, enhanced the anticancer activity of chemotherapy drugs [30,31]. This suggests that the type of interaction between isothiocyanates and 5-FU depends on the aggressiveness and hormone status of the cell line, with this interaction being more beneficial in hormone-resistant, aggressive cancer cells. This finding could be beneficial when considering an alternative option for the efficient treatment of such cancers. 

### 2.2. Mechanism of Interaction

In the next stage our study, we tested the mechanisms involved in the promising interactions (additive or synergistic) through the evaluation of cytostatic and cytotoxic effects (Figure 3). Since cell growth (measured using the MTT assay as shown in Figure 2) may be inhibited by cell cycle arrest or cytotoxicity, i.e., cell death/necrosis, describing the profile of these effects is important for the full and reliable characterization of the interaction mechanism. We investigated the effect of combined treatments at fa = 0.75, which reduced cell growth by approximately 75% in comparison to the control. They exerted a synergistic effect in HT-29 and Caco-2 colon cancer cells and an additive effect in PC-3 prostate cancer cells (Figure 2). Additionally, the results of the individual treatments were quantified.

First, we examined the morphological changes (Figure 3A–C). The most potent results were observed after the combined treatment, which included a decrease in cell number and significant morphological changes—detached shrunken cells indicative of cell death. 

The flow cytometry method was further applied to assess the cytostatic (cell cycle distribution) and cytotoxic effects (the number of living cells in the culture) of the combined and individual treatments (Figure 3D–F). In colon cancer cells, the accumulation of cells in the S phase of the cell cycle was observed after 5-FU treatment and the combined treatment, and no statistically significant differences between 5-FU and the combined treatment were observed. The effect was most prominent in the HT-29 cells: after both types of treatment, 80% of the cells were in the S phase of the cell cycle, which is four times more than in the case of the control cells. In turn, in the Caco-2 cells, the percentage of cells after 5-FU treatment and after the combined treatment amounted to around 50–60% in the S phase of the cell cycle. These results indicate that the cytostatic effect of the combined treatment, i.e., cell growth arrest in the S phase (typical for 5-FU), is a result of 5-FU only. 

Many authors highlighted the impact of a strong cell cycle block on the induction of synergistic cytotoxic effect. Some claimed that the block in the phase, typical for an anticancer drug, is crucial for the occurrence of a synergistic interaction between anticancer drugs and natural compounds. It was proven, for example, in resveratrol treatment with 5-FU in HT-116 colon cancer cells, in doxorubicin treatment in B16 melanoma cells, and in the case of cisplatin cotreatment in bladder cancer [32,33,34]. This was also shown in our previous studies, which were conducted on HT-29 cells after sulforaphane and 5-FU treatment [22]. In this study, in both colon cancer cell lines, only 5-FU altered the cell cycle distribution, and this effect was fairly strong. The same changes were observed after the combined treatment. We observed that combined 5-FU and 2-oxohexyl ITC treatment induced a synergistic cytotoxic effect. The decrease in the level of viable cells after the combined treatment in the colon cell culture was significantly lower than in the control cells (a 40% drop), but it was also lower in a statistically significant manner than after the individual 5-FU and 2-oxohexyl ITC treatments. A more prominent effect of combined treatment was observed in the HT-29 cells since in case of these cells no effect was observed after the individual treatments, while in the Caco-2 cells, the individual treatments induced a statistically significant drop in the ratio of living cells in the culture.

On the contrary, in PC-3 prostate cancer cells, both tested compounds altered the cell cycle distribution. The number of cells in the S phase after the 5-FU treatment grew eight times in comparison to the control cells (up to 80%). Treatment with 2-oxohexyl ITC alone induced a block in the G2/M phase of the cell cycle, and approximately 50% of the cells were observed to be in this phase of the cell cycle. After the combined 5-FU and 2-oxohexyl ITC treatment, we observed cell accumulation in both phases of the cell cycle: G2/M and S; however, the effects were weaker than after the individual treatments. We observed 25% of the cells to be in the S phase of the cell cycle and 50% of cells to be in the G2/M phase of the cell cycle. In comparison to colon cancer cells, the additional effect of the sulforaphane analog on cell cycle distribution was observed. Similar results were reported by González-Sarrías et al. who showed that due to the influence of urolithin A and 5-FU on different cell cycle phases in HT-29 cells, the interaction of the compounds was additive [35]. At the same time, the results of cytotoxic studies revealed that the drop in the number of living cells in the culture after the combined treatment was the same as after the 2-oxohexyl ITC treatment alone, which indicates that the cytotoxic effect is the result of the 2-oxohexyl ITC activity only.

To summarize these results, the combined 5-FU and 2-oxohexyl ITC treatment exhibited higher effectiveness in colon cancer cells. The observed synergistic interaction between these two compounds is the result of strong blocking of the cell cycle caused by 5-FU and a drop in the living cell ratio in the culture, which is the result of synergistic enhancement of the 5-FU and 2-oxohexyl ITC activities alone; this is also reflected in the cell culture images. As shown in Figure 3, the drop in the percentage of living cells is associated with the occurrence of apoptosis. The more prominent proapoptotic effect is observed in the more aggressive HT-29 cells. In PC-3 prostate cancer cells, the additive effect of the combined treatment is the result of a 2-oxohexyl ITC-induced drop in the percentage of living cells and the effect of the two compounds on different cell cycle phases.

### 2.3. Apoptosis Studies

Clinically, apoptosis plays an indispensable role in the eradication of tumor cells through their death. Therefore, apoptosis was the expected effect of the combination treatment [36]. To investigate the apoptosis mechanism, we tested caspase activity using the fluorometric assay. Moreover, we investigated the changes in procaspases and the resulting cleaved caspase protein levels using the Western blot method. We included caspases 8 and 9, which are known as initiator caspases, and caspase 3, which is considered to be an effector caspase. They are characteristic of apoptosis induction via isothiocyanates or via 5-FU in HT-29 cells [37,38]. The tests were conducted after 24 h and 72 h of treatment. Incubation time of 24 h is generally used in caspase activity assays and incubation time of 72 h is generally used in the research of isothiocyanate interaction with 5-FU in cancer cells [19,22]. 

**Figure 4:**
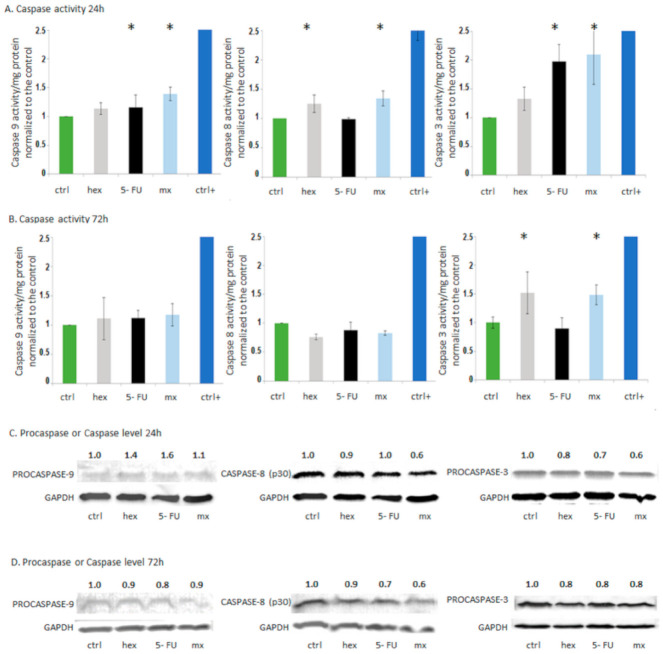
Apoptosis studies. (**A**) Caspase (Caspase 9, Caspase 8, and Caspase 3) activity/mg protein normalized to the control (ctrl) after the combined 5-FU and 2-oxohexyl ITC treatment, individual 5-FU treatment, individual hex treatment, and positive control treatment (ctrl+) after 24 (**A**) and 72 h incubation (**B**). * Statistically significant difference from the control; *p* < 0.05. Changes in the levels of apoptosis-related proteins obtained by Western blotting after the administration of individual compounds, the combined treatment of 5-FU with hex, and for the control cells (ctrl) after 24-h incubation (**C**) and after 72-h incubation (**D**). Glyceraldehyde-3-phosphate dehydrogenase (GAPDH) was amplified as an internal control. The results of the bands of procaspase forms were quantified by densitometric analysis, and their intensity was normalized with respect to GAPDH.

After 24-h incubation with the combined treatments, the activity of caspase 8 and caspase 9 increased by approximately 30% in comparison to the control cells (Figure 4). The activity of caspase 8, which is an indicator of receptor pathway apoptosis, was found to be at the same level as after the 2-oxohexyl ITC incubation; the activity of caspase 9, the hallmark of the mitochondrial intrinsic pathway of apoptosis, was found to be at the same level as after the 5-FU treatments. The effector caspase 3 activity increased after both tested incubation times. After 24 h, the combined treatment increased the caspase 3 activity by approximately 100% in comparison to the control cells and in the same way as after the 5-FU treatments. After 72 h, we observed an increase in the activity of effector caspase 3. It was at the same level as after the 2-oxohexyl ITC incubation and demonstrated an approximately 50% increase in comparison to the control cells. These results indicate that the increase in effector caspase activity is the combined result of the effects of 5-FU and 2-oxohexyl ITC. While 5-FU induces the mitochondrial pathway, 2-oxohexyl ITC induces the receptor-mediated pathway of apoptosis. Our findings correlate with those described in the literature. The receptor pathway is considered to be the main apoptosis mechanism of isothiocyanate activity and combined anticancer drug treatments. The apoptosis receptor pathway induction by the combined treatment with isothiocyanate and oxaliplatin was demonstrated by Kaminski et al. [37]. Our earlier studies in HT-29 cells showed synergistic caspase 8 activation by sulforaphane and 5-FU [22]. On the other hand, an induction of the mitochondrial pathway of apoptosis by 5-FU in colon cancer cells was previously demonstrated by Mhaidat et al. [38]. Our findings revealed that isothiocyanate combined with 5-FU can simultaneously induce the intrinsic and extrinsic pathways of apoptosis in aggressive colon cancer HT-29 cells. Interestingly, our results indicate that effector caspase 3 is activated at different timepoints by 5-FU and 2-oxohexyl ITC. While 5-FU-mediated induction of the intrinsic apoptosis pathway results in the increased activity of caspase 3 after 24 h, 2-oxohexyl ITC, in turn, requires a longer time (72 h) to induce caspase 3 activity. These results indicate that the 2-oxohexyl ITC-induced caspase 3 activity is the result of the activation of procaspase 8 or, considering the lack of observed activity of caspase 8 after 72 h, another signaling pathway may be induced by this compound, which leads to the induction of caspase 3. 

In addition to caspase activities, their full-length (not active) and cleaved (active) levels were studied by Western blotting. We were not able to visualize the active form of caspase subunits. In the case of caspase 8, we detected its C-terminal cleavage product, i.e., subunit p30, which represents an alternative sequence of cleavage events and, as shown by Hoffmann et al., it can sensitize cells toward death receptor-induced apoptosis [39]. The studies showed a decrease in the level of subunit p30, mainly after 24 h and 72 h of the combined treatment. 

The procaspase 9 level significantly increased after 24 h of 5-FU treatment, which may be the result of increased transcription or inhibited ubiquitination and was shown to trigger caspase 9 activation and intrinsic apoptosis [40]. The level of procaspase 3 decreased after the individual 5-FU treatment and the combined treatment, which indicates that its activation corresponds with caspase activity. The results for 2-oxohexyl ITC are inconsistent in terms of the differences in the levels of procaspase and caspase activity. This indicates that 2-oxohexyl ITC may change the catalytic activity of caspases. As discussed by Parrish et al., the activities of caspases can be regulated both by the cleavage process and the expression intensity, and by post-translational modifications and protein/protein interactions [41]. As discussed above, the induction of another signal transduction pathway by 2-oxohexyl ITC should also be taken into consideration as a mechanism of apoptosis induction in HT-29 cells.

### 2.4. Autophagy Studies

Many researchers emphasize that studies of interaction mechanisms should not only include cell growth or apoptosis experiments, but also nonapoptotic cell death studies. Although autophagic cell death is the most investigated form of nonapoptotic cell death, it is a disputed process that triggers (depending on its intensity) two opposite effects: cytoprotective or cytotoxic. In the first stage, autophagy is a cytoprotective process that can be induced by oxidative stress, nutritional stress, or chemical reactions; however, an increase in the intensity of the process leads to cell death [42].

The autophagy experiments were based on the studies of the LC3-II protein, which is regarded as an autophagy marker [43,44]. In cells, LC3 occurs in two isoforms: LC3-I (the cytosolic form) and LC3-II (the autophagic vacuole membrane-bound form). To detect the LC3-II protein, microscopic techniques and the Western blot method were used. 

**Figure 5:**
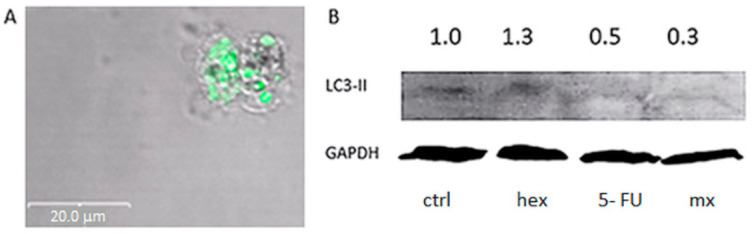
Autophagy detection: visualization of autophagic vacuoles in cells after the combined treatment with 2-oxohexyl ITC (hex) and 5-FU (**A**). Protein expression of LC3-II. GAPDH was a loading control. Protein levels were evaluated by the Western blot method (**B**).

Both experiments showed that only after the incubation of cells with 2-oxohexyl ITC, green LC3-II protein clusters in the autophagic vacuoles (Figure 5A) and an increase in the level of LC-II were observed (Figure 5B). In the present study, we first demonstrated that 2-oxohexyl ITC, like its parent molecule, is an inductor of autophagy. Interestingly, the level of LC3-II dropped after the combined treatment in comparison to the 2-oxohexyl ITC treatment alone. This proved that the autophagy process was not induced by the combination of 5-FU and 2-oxohexyl ITC; this is contrary to our earlier experiment which showed that 5-FU applied with sulforaphane can trigger a synergistic anticancer effect in breast cancer cells (among others) via autophagic cell death [19]. This indicates that the exchange of sulfur to carbon in the sulforaphane molecule changes the molecule’s activity in such a way that it is not capable of inducing autophagy in combination with 5-FU.

## 3. Conclusions 

The study of the interaction between 5-FU and the sulforaphane analog, 2-oxohexyl ITC, showed that this combination is more effective in more aggressive types of cancer (as typified by colon and prostate cancer cell lines). The synergistic activity was shown in colon cancer cells and the most beneficial effect was observed in HT-29 cells. The strong block in the S phase of the cell cycle induced by 5-FU and the simultaneous synergistic drop in the ratio of living cells induced by the combined treatment were shown to underlie the synergistic interaction between 5-FU and 2-oxohexyl ITC. The process of apoptosis, rather than through autophagy, was induced by this combination. By comparing the signaling pathway with that induced by the combination of 5-FU and the parent molecule, sulforaphane, our results indicate that both the receptor (activation of caspase 8 by 2-oxohexyl ITC) pathway and the mitochondrial (activation of caspase 9 by 5-FU) pathway are involved in the apoptosis process in HT-29 cells, which makes this combination a considerable candidate for a combined colon cancer treatment.

## 4. Materials and Methods

### 4.1. Cells and Reagents

Colon cancer cell lines (Caco-2 and HT-29) and prostate cancer cell lines (LNCaP and PC-3) were obtained from the American Type Culture Collection (ATCC, Manassas, VA, USA). Colon cancer cells were grown in the MEM (Cytogen, GmbH Bienenweg, Berlin, Germany). Prostate cancer cells were grown in RPMI (Cytogen). The media were supplemented with 20% (Caco-2) or 5% (HT-29, PC-3, LNCaP) fetal bovine serum (Gibco, Grand Island, NY, USA), 1% antibiotic solution (10,000 U/mL penicillin and 10 mg/mL streptomycin), 25 µg/mL amphotericin B (Sigma-Aldrich, St. Louis, MO, USA), and 1% nonessential amino acids (Sigma-Aldrich). 

All the cell lines were harvested at 37 °C in a humidified incubator with a 5% carbon dioxide atmosphere. The cells were routinely tested for the presence of mycoplasma using polymerase chain reaction (PCR) detection and microscopic examination [45].

While 5-FU was obtained from Sigma-Aldrich, 2-oxohexyl ITC was synthesized as described previously by Schmidt and Karrer [46]. 

### 4.2. Cell Growth Assay

The cells were incubated in a 96-well plate at a density of 4 × 10^4^ cells per ml. Increasing concentrations of 5-FU and 2-oxohexyl ITC were added to cells in the logarithmic phase of growth; incubation lasted for 24 or 72 h. The cell growth was evaluated using the MTT (3-(4,5 dimethylthiazol-2,5-diphenyl tetrazolium bromide) assay. At the end of incubation, the medium was removed and 0.25 mg/mL MTT (Sigma-Aldrich) was added. The absorbance of formazan was measured at a wavelength of 570 nm with background subtraction at 690 nm. The microplate scanning spectrophotometer (PowerWave X, BioTek, Winooski, VT, USA) was used for the measurements [24].

### 4.3. Quantitative Analysis of Interactions

To investigate the combined effect of 2-oxohexyl ITC and 5-FU, the Chou-Talalay method was used [26]. In the combination treatment studies, the tested substances were added in concentrations that were approximately their IC50 multiplicity. Sequential scheme of cell treatment was applied in case of each cell line. The sequential scheme involved a 24-h treatment with 2-oxohexyl ITC followed by treatment with 5-fluorouracil for 72 h. In addition, in the same experiments, the cells were incubated with each of the substances alone at concentrations corresponding to the concentrations used in the combined treatment. The effect was estimated with the MTT assay. In order to define the type of effect, the combination index (CI) was calculated.

The CI values were calculated according to the formula for mutually exclusive drugs: CI = (D)1/(Dx)1 + (D)2/(Dx)2, where (Dx)1 and (Dx)2 denote the concentrations of tested substances 1 and 2 used in the single treatment that were required to decrease the cell number by x%; and (D)1 and (D)2 denote the concentrations of tested substance 1 in combination with substance 2 that together decreased the cell number by x%.

The value of the combination index (CI) > 1.1, < 1.1, and from 0.9 to 1.1 indicates antagonism, synergism, and additive effect, respectively. CI and DRI were calculated using CompuSyn (ComboSyn, Paramus, NJ, USA).

### 4.4. Mechanism Investigation

The mechanism of interactions was investigated at the value of the percentage (fraction) of cells whose growth was affected (fa) = 0.75. Values of the tested concentrations causing fa 0.75 were calculated using CompuSyn (ComboSyn, Paramus, NJ, USA). The assessment included sequential treatments and the administration of substances alone at concentrations corresponding to the concentrations used in the combination treatment.

#### 4.4.1. Cell Cycle Analysis

The cytostatic effects of the 5-FU treatment, 2-oxohexyl ITC treatment, and their combined administration were tested. After incubation, the cells were trypsinized and rinsed with PBS and suspended in 75% ice-cold ethanol. Each sample was stained with a staining solution containing 50 µg/mL propidium iodide (PI) (Sigma-Aldrich), 100 µg/mL RNase (Sigma-Aldrich), and 0.1% Triton X-100 (Sigma-Aldrich). The test determined the content of DNA, and according to the results, the phase of the cell cycle was defined. Cell cycle distribution was examined with a FACS Calibur flow cytometer (BD Biosciences, San Jose, CA, USA), and CellQuest (BD Biosciences). The cell cycle was analyzed with Multi Cycle AnalysisTM (Phoenix Flow Systems, San Diego, CA, USA) [47].

#### 4.4.2. Cytotoxic Effect Determination

The cytotoxic effects of 5-FU administration, 2-oxohexyl ITC administration, and their combined administration were examined by calculating the number of living and dead cells. Fluorescein diacetate (FDA) and (PI) were used to stain live and dead cells, respectively. The cells were trypsinized and rinsed with PBS. Each 100-µL sample in PBS was treated with 10 µL of 6.25 g/mL fluorescein diacetate (Sigma-Aldrich) and 10 µL of 50 µg/mL PI (Sigma-Aldrich). After incubation on ice, 0.5 mL PBS was added and the sample was analyzed with a FACS Calibur flow cytometer. Cell viability was calculated using WIN MDI2.9 (Becton-Dickinson, Franklin Lakes, NJ, USA) [24].

#### 4.4.3. Microscopic Identification of Apoptosis

For the purposes of microscopy analyses, the cells were stained with fluorescein isothiocyanate (FITC) Annexin V Apoptosis Detection Kit I (BD Biosciences Company, San Jose, CA, USA). The cells were stained with Annexin V-FITC (5 µL per 1 mL buffer) and PI (5 µL per 1 mL buffer), and fluorescence was excited with 488 nm and 543 nm lasers, respectively. The FITC and PI fluorescence values were collected with the help of a confocal microscope (Olympus, Shinjuk, Tokyo, Japan) using 520-nm and 600-nm BP filters, respectively. Living cells (AnnV−/PI−), early apoptotic cells (AnnV+/PI−), and necrotic or late apoptotic cells (AnnV+/PI+) [48] were distinguished.

#### 4.4.4. Microscopic Identification of Autophagy

Microtubule-associated proteins 1A/1B light chain 3B (LC3-II) was detected using Premo™ autophagy sensor LC3-II-GFP according the manufacturer’s protocol. After 24 h, the cells were sequentially treated for 48 h. The LC3-GFP proteins were visualized using an Olympus FV500 confocal microscope using the appropriate filters for GFP (488/520 nm) [49].

#### 4.4.5. Caspase 3, 8, and 9 Fluorometric Assays

The caspase 3, 8, and 9 fluorometric assays were used to evaluate the activity of caspases after the 5-FU treatment, 2-oxohexyl ITC treatment, and combined treatment. Briefly, 10^6^ cells were seeded in 75-cm^2^ flasks and treated as previously described. As a positive control, 100 µM etoposide was used. After the treatment period, the cells were trypsinized and 3 × 10^6^ cells were used per assay. The test was performed according to the manufacturer’s protocol (BioVision). Fluorescence was measured at 400 nm excitation and 505 nm emission wavelengths using an Infinite M1000 Pro (Tecan) microplate reader. The obtained results were normalized to the protein level in cell lysates. The protein level in cell lysates was measured using the BCA test [50].

#### 4.4.6. Western Blot Assay

The levels of proteins engaged in the apoptotic process (caspase 8, procaspase 9, and procaspase 3) and autophagy, i.e., LC3-II, were tested. A sample was separated with SDS-PAGE and transferred to a PVDF membrane (Amersham, GE Healthcare Life Sciences, Freiburg, Germany) using vertical electrophoresis apparatus Mini-PROTEAN^®^ 3 Cell (BioRad, Hercules, CA, USA). The proteins were immunoblotted with the primary monoclonal antibodies: anti-caspase 8, anti-glyceraldehyde-3-phosphate dehydrogenase (GAPDH) (Thermo Fisher Scientific, Waltham, MA, USA), and anti-caspase 3 and 9 (Cell Signaling, Leiden, The Netherlands). GAPDH was amplified as an internal control. Protein bands were visualized with a Bio Imaging System (DNR Lumi BIS, Jerusalem, Israel) using the fluorescent method of a WesternDot kit (Thermo Fisher Scientific). Protein bands were characterized using the ImagineR (Sun Microsystems, Santa Clara, CA, USA) analysis software [50].

## Data Availability

Not applicable.

## Sample Availability

Samples of the compounds are not available from the authors.
